# Toward understanding the neurophysiological basis of peripersonal space: An EEG study on healthy individuals

**DOI:** 10.1371/journal.pone.0218675

**Published:** 2019-06-24

**Authors:** Antonino Naro, Rocco Salvatore Calabrò, Gianluca La Rosa, Veronica Agata Andronaco, Luana Billeri, Paola Lauria, Alessia Bramanti, Placido Bramanti

**Affiliations:** Neurorehabilitation Unit, IRCCS Centro Neurolesi Bonino Pulejo, Messina, Italy; University of Ontario Institute of Technology, CANADA

## Abstract

The subcortical mechanisms subtending the sensorimotor processes related to the peripersonal space (PPS) have been well characterized, whereas less evidence is available concerning the cortical mechanisms. We investigated the theta, alpha and beta event-related spectral perturbations (ERSP) while holding the forearm in different positions into the PPS of the face. Fifty healthy individuals were subjected to EEG recording while being provided with median nerve electric stimulation at the wrist of the right hand held at different hand-to-face distances. Theta and beta rhythms were significantly perturbed depending on the hand-to-face distance, whereas alpha oscillations reflected a more general, non-specific oscillatory response to the motor task. The perturbation of theta and beta frequency bands may reflect the processes of top-down modulation overseeing the conscious spatiotemporal encoding of sensory-motor information within the PPS. In other words, such perturbation reflects the continuous update of the conscious internal representations of the PPS to build up a purposeful and reflexive motor response.

## Introduction

The brain processes environmental information depending on their origin—that is, whether they arise from the body or the extrapersonal or peripersonal space (PPS). The latter refers to “the nearby representational space in terms of what was reachable—that is, within range of the arm’s reach” [1].

Multisensory information arising from the PPS is processed together to build an internal spatio-temporal reconstruction of the neighboring environment with a body part-centered frame [2]. An example of these sensory processes is represented by visuo-tactile integration between tactile stimuli, provided on a surface of the body, and visual stimuli, presented close to the stimulated body surface [3]. The internal spatio-temporal reconstruction of the PPS allows (i) building up purposeful motor behaviors aimed at interacting with objects and persons, e.g., to grasp food and useful objects, and (ii) controlling the subcortical networks (namely, top-down modulation) generating reflex responses to avoid threats near the body, e.g., to avoid a bee flying towards the face [3–17]. To internally reconstruct the PPS, it is required combining information on the spatial properties of the environment with the information on the current capacity to act [18].

The neuroanatomical substrates of such internal spatio-temporal reconstruction and of the PPS-related motor plan generation have been partially characterized in humans using transcranial magnetic stimulation and fMRI [19–25]. These studies identified a PPS-related complex network encompassing ventral premotor, intraparietal, occipital and somatosensory areas through which the location and timing of multisensory stimuli are coded into body part-centered PPS coordinates. Indeed, perceiving objects in the PPS activates a neural network largely overlapping the one subtending voluntary motor action and motor imagery [18]. Furthermore, the boundaries of PPS are continuously tuned depending on body part movements and positions; last, they are flexible because they adapt to experience [3,5,15,26–27].

On the other hand, the neurophysiological basis subtending PPS functions is still only partially understood. It has been suggested that the subcortical circuits (brainstem, spinal cord) mediating PPS-related responses undergo a top-down modulation from higher-order cortical areas depending on many factors, including the position of the hand toward the PPS [24–25,28–32].

This top-down modulation has been estimated by measuring the changes in the magnitude of several brain responses (including fMRI BOLD signals, event-related potentials, and the hand-blink reflex) to various distances between a target and the PPS of the face during multisensory or sensorimotor paradigms [33–37]. Specifically, the magnitude of the evoked brain response has been found to be proportional to the distance of the target, e.g., the hand, from a body surface, e.g., the face [38–40], in keeping with the temporal rules of multisensory integration [17,24,28–32].

Indeed, bimodal stimulation at the same location in the PPS or in the same hemispace to tactile stimuli elicits greater brain responses compared to the algebraic sum of the responses to unimodal single stimuli, even within the same location in the PPS or in the same hemispace to tactile stimuli [28,31]. This distance-dependent response is also suggested by the sensitivity of the extinction phenomenon (i.e., the inability to perceive multiple stimuli of the same type delivered simultaneously) to the body-target distance (e.g., face-hand) in patients with right brain damage [41–43].

A still debated issue is the degree of awareness related to PPS processing [44]. The knowledge of PPS related-awareness may be important to better understand the neurophysiological basis of neurological or neuropsychiatric disorders involving the PPS, including neuropsychiatric disorders [45], cognitive decline [46], and chronic Disorders of Consciousness [47].

The fact that PPS partially activates a neural network overlapping with those subtending voluntary motor action and motor imagery and, partially, consciousness generation and maintenance [21,48] would support the hypothesis of consciousness of the PPS. Indeed, the activation of the PPS network may presuppose as many different levels of awareness as different the aspects of the sensorimotor processes related to PPS are, including attention (top-down control), target position within the PPS, threatened body area, cognitive expectation about the stimulus, potential cross-modal link between different stimuli, the spatial extent of PPS (the amplitude of PPS-related responses is modified if sight is somehow limited), the social content, the functional consequences of sensory information (i.e., protective and goal-directed responses), and the cortico-subcortical control of the brainstem and spinal circuitries mediating PPS-related reflex responses [3,5,49–54].

A suitable way to assess awareness related to PPS may be represented by EEG analysis. A few studies investigated brain oscillations elicited during multisensory tasks perturbing the PPS [55–56]. It has been shown that the motor coding of visual objects within the PPS is expressed in the alpha band (8–13 Hz), depending on the object location and the goal of the motor task. However, partial knowledge is available on the changes of sensory-motor rhythms during PPS-related motor tasks. Studying these rhythms and, in particular, their top-down modulation may offer useful information concerning PPS-related movement planning and execution and their level of awareness, as specific modulation of brain responses evoked by PPS perturbation would suggest awareness of the PPS [46–47,57–59]. To this end, we investigated the spatial rules of a visuo-tactile interaction task targeting the PPS of the face. We employed the paradigm of hand-blink reflex elicitation described by Sambo and Iannetti [52–53]. Specifically, we estimated the effects of some spatial configurations (i.e., different hand-to face-distances) on the magnitude of brain response measured by event-related spectral perturbation (ERSP) in a sample of healthy individuals. ERSP coincides with the event-related dynamics of the EEG spectrum induced by, but not phase-locked to, the onset of a stimulus; thus, ERSP reveals aspects of event-related brain dynamics not contained in the ERP average of the same response epochs [60–61]. We focused on theta, alpha, and beta ERSP as these may reflect diverse sensorimotor processes under dissimilar spatial configurations, i.e., diverse hand-to-face distance [62–64].

## Materials and methods

### Participants

Fifty healthy right-handed volunteers (31 males and 19 females), aged between 23 and 45 years (33±5 years), were enrolled in this study. Only self-reported right-handed subjects were included in the study to avoid a handedness confound. Moreover, the handedness was assessed by the Edinburgh Inventory [65], and only subjects with a high, positive scoring (suggesting strong right-handedness) were recruited. Last, subjects were excluded from the study if information from a standardized questionnaire suggested the possibility of any neurological disorder, particularly perinatal asphyxia or kernicterus, head trauma, loss of consciousness, epileptic seizures, meningitis or encephalitis, or delayed or disturbed language development. All procedures performed in studies involving human participants were in accordance with the ethical standards of the institutional and/or national research committee and with the 1964 Helsinki declaration and its later amendments or comparable ethical standards. The local Ethics Committee of the IRCCS Centro Neurolesi Bonino Pulejo (Messina, Italy) approved the study. Informed consent was obtained from all individual participants included in the study.

### Experimental design

**Fig 1:**
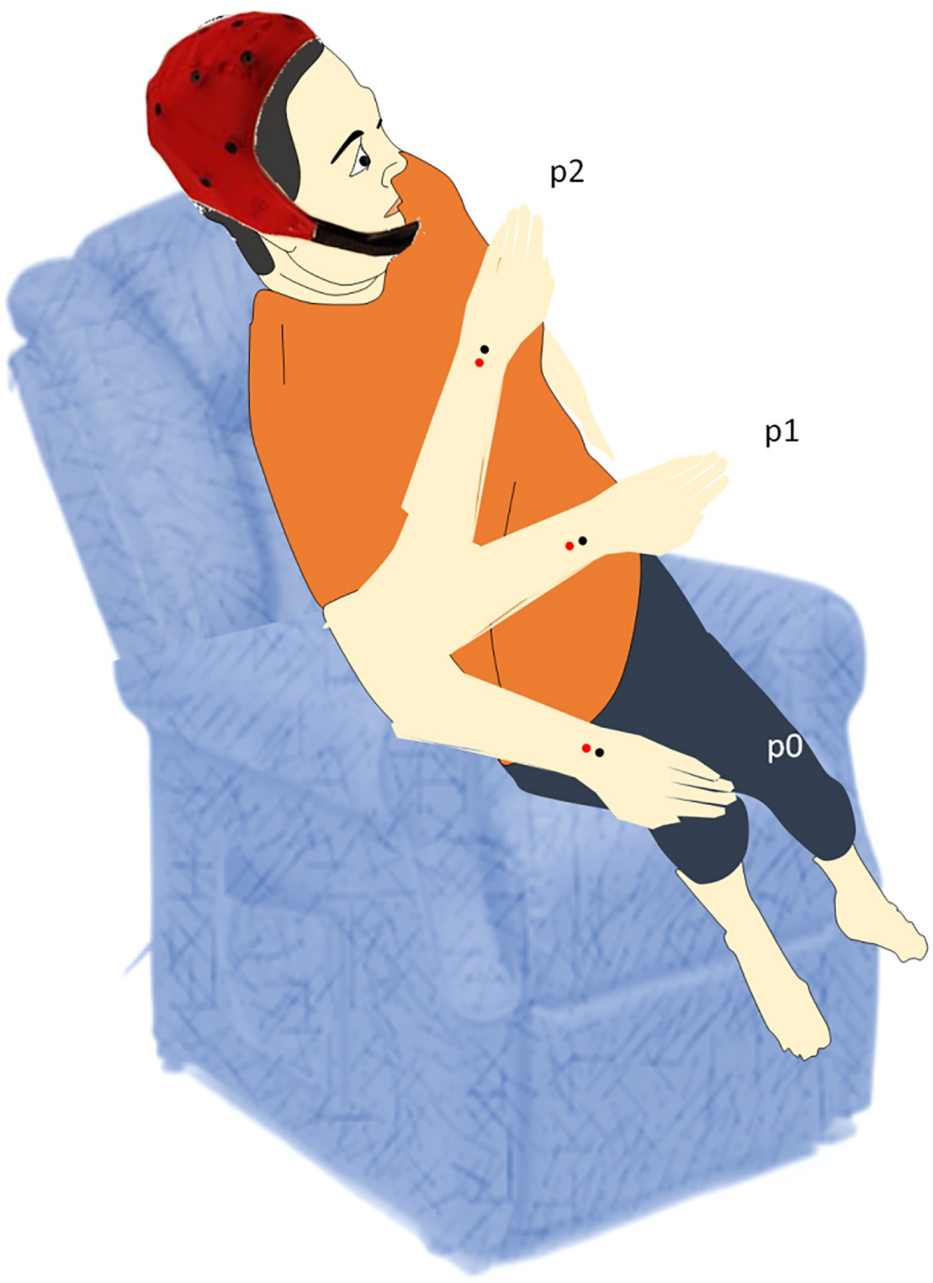
Experimental design.

Subjects were sitting in a comfortable armchair reclined at 45°, in a quiet and mildly lit room with the right hand put at rest on the ipsilateral thigh, the palm facing up, and the eyes open. They were asked to look straight forward at a point marked on a wall. The subjects were invited (verbally by the experimenter) to hold the right forearm for 20 seconds at a pre-established distance between the hand and the face. We employed the ultrafar position, p0, with the hand lying on the thigh; the far position, p1, with the hand held between the thigh and the face; and the near position, p2, with the hand held at ~4cm from the face (Fig 1).

Thus, the hand was visible by the subject only in the far and near positions, as the thigh was out of sight (Fig 1). The order of the positions to be held was random; the participants were instructed on how to hold the right forearm and which were the positions to be held through a video before starting the experiment.

While holding the hand in a position, the subject was provided with a single brief electric stimulus (square wave pulse) at the median nerve at the right wrist [66–67], between 8 to 12 seconds after having started to hold the position. The square wave pulses were applied to the median nerve at the right wrist using a bar-electrode (located slightly medial and proximal to the styloid process of the radius and just lateral to the tendons of the wrist) wired to a constant-current stimulator (Digitimer D-160 stimulator; Digitimer Ltd, Welwyn Garden City, Herts, UK). The cathode was located proximally and the pulse width was 500 μs. The stimulation intensity was set at 1.5 times the individual sensory threshold (defined as the minimum stimulation intensity resulting in sensation in the vicinity of the disk electrodes in at least 5 out of 10 consecutive trials with the hand kept at rest), tested by tuning the stimulation intensity in ±0.5mA steps. Thus, the subject was invited to move and then hold the upper limb in another hand-to-face position (randomly among p0, p1, and p2, avoiding position repetition). Each position was repeated 50 times in a random order during the experiment while recording the EEG. Participants were required to count mentally the stimuli they received, given that attention can interfere with brain plasticity also when employing repeated nerve stimulation (Stefan et al. 2004).

In a separate control session (in a different day), 25 healthy individuals, age- and gender-matched to the main group, were subjected to a different motor task while recording EEG. Specifically, the forearm positions to be held (paired with the same brief electric stimulus at the median nerve at the right wrist) were toward and away from the thigh in a horizontal plane (at 90°, 45°, and 0° from the thigh). This control experiment was necessary to control for holding the arm in various positions of equal distance away from the face and to control for focus and attention. Further, it is known that the brain elaborates sensorimotor information in a different way whether they come from the extra-personal space or PPS, and depending on the direction of target movement toward and away from a body part [7,68–71]. Thus, it is mandatory to check for different levels of awareness whether the stimulus falls within the extra-personal space or PPS, and depending on the cognitive demand for the proper motor output generation (behavioral or defensive).

### EEG recording and analysis

EEG was continuously recorded during the experiment using a BrainQuick System (Micromed, Mogliano Veneto, Italy) equipped with a 21-channel cap. EEG data in microvolts (μV) were sampled at 512Hz, referenced to the linked mastoids (checking for impedance asymmetry), and filtered at 0.3–45Hz. EEG was segmented into 12s epochs, [-6:6]s with respect to the electric stimulus provision (0 time). The electric stimulus provision occurred at each of the hand positions, which was changed 50 times during the experiment. Thus, 150 epochs were obtained (50 for each of the hand position). Trials contaminated by eye blinks (i.e., Fp1 or Fp2 exceeding ±60μV relative to baseline), EOG movements (exceeding ±30μV relative to baseline as measured by flat electrodes placed on the outer canthi of the eyes, under the right eye and over the ear lobes to capture any eye movements and blinks), or other artefacts within 800ms after electric stimulus provision (a voltage exceeding ±60μV relative to baseline), were excluded from the analysis. Further, Independent Components Analysis (ICA) was used to identify and reject artefactual trials.

Then, about 125±7 artifact-free EEG segments were used to calculate the spectral energy on pooled electrodes (frontal, pooled across Fp1,F3/7,Fz and Fp2,F4/8,Fz; centroparietal, C3/P3,Cz and C4,P4,Pz; temporal, T3/5 and T4/6, and occipital, O1 and O2) and frequency bands (theta 3.5–8Hz, alpha 8–12Hz, and beta 12–30Hz), using a sinusoidal wavelet transform [72]. The energy was extracted for and log-transformed on each trial separately. Time-frequency decomposition was applied to all the segmented data, comparing the energy of the mean pre-stimulus [-4:0]s to the mean post-stimulus period [0:6]s. A paired t-test was applied for each frequency band on the pooled electrodes. By averaging and baseline-correcting these trials to [-6:-4s], we obtained the ERSPs [73].

Owing to the exploratory nature of our study, we focused on the general tendency in the post-stimulus oscillation pattern (that is, the increases and decreases of the energy around each region of interest) rather than the precise timing of the differences. So, we adjusted a 2-degree polynomial regression on the baseline-corrected [0:6]s spectral energy of each trial separately for the three frequency bands using least-squares.

### Statistical analysis

Hand-position effect on each frequency-band ERSP was tested using paired t-tests (one-way repeated measures ANOVA) with 2000 permutations and false discovery rate correction for multiple comparisons. Hand-position effect on ERSPs was assessed using unpaired t-tests. Statistical significance was set at p<0.05. Then, we extracted three features for each trial and frequency band: the intercept (the baseline corrected energy at 3 sec post-stimulus), the slope (the linear tendency of the post-stimulus energy), and the quadratic effect (signaling a U-shape or its inverse for a positive or, respectively, negative coefficient). Each of the six (dependent) variables obtained (*feature* × *frequency band*) was subjected to an ANOVA with *electrode pool* (four levels) and *hand-to-face distance* (three levels) as factors, thus getting 72 values per trial. Statistical significance was set at p<0.05. *Post*–*hoc* paired *t*–tests adjusted for multiple comparisons with the Bonferroni method were thus performed.

## Results

**Fig 2:**
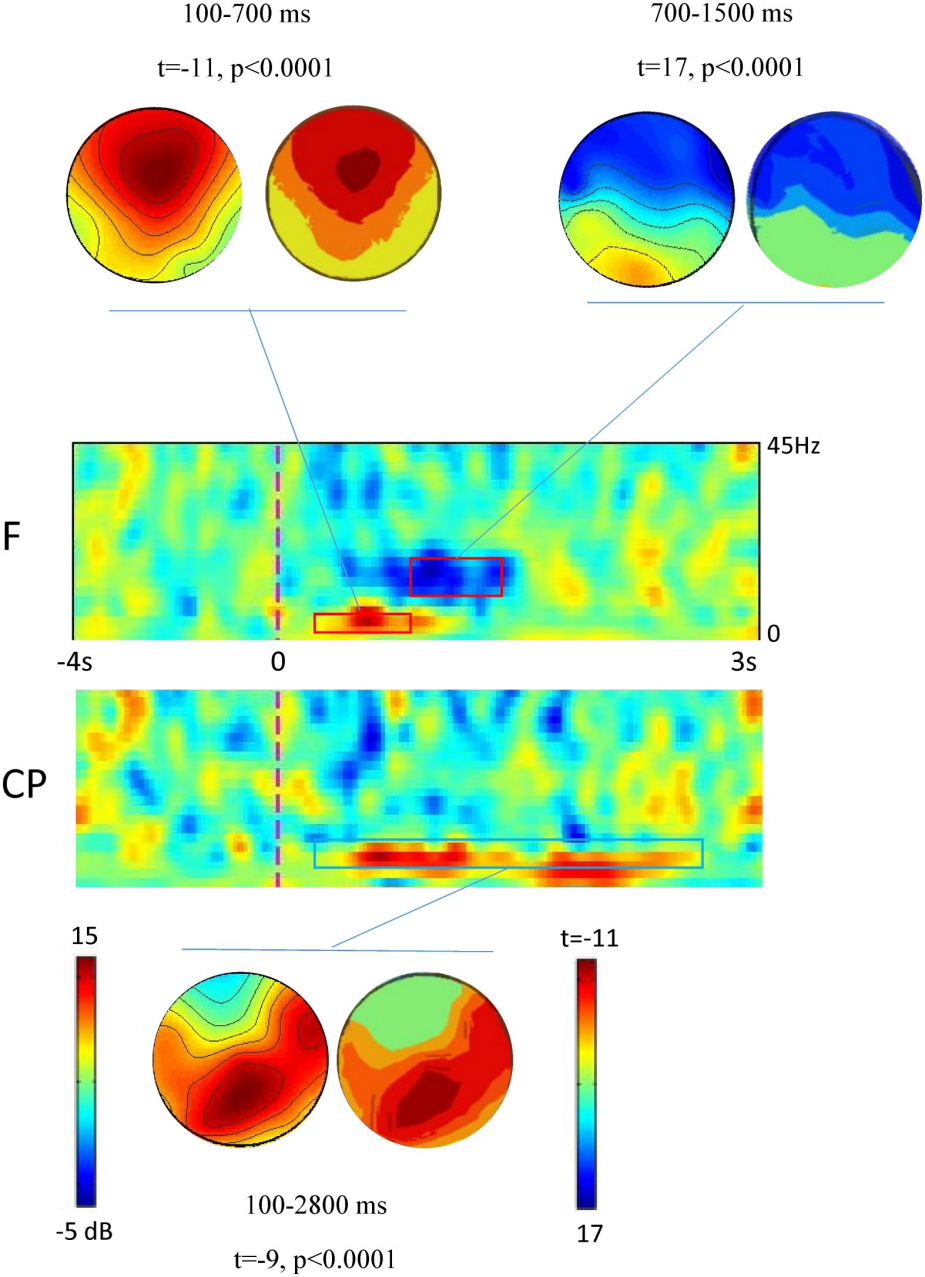
ERSP plots of pooled electrodes in frontal and centroparietal scalp sites. Electric stimulus onset is at 0ms. Pairwise headplots indicate the left the ERSP topography at specific periods, the right the two-dimensional grand mean *t-*maps (negative *t-*test value indicates a post-stimulus ERSP increase).

All the participants performed the task without any difficulty. Post-stimulus spectral changes were largely visible (Fig 2). ERSP data are enclosed in S1 Table.

**Fig 3:**
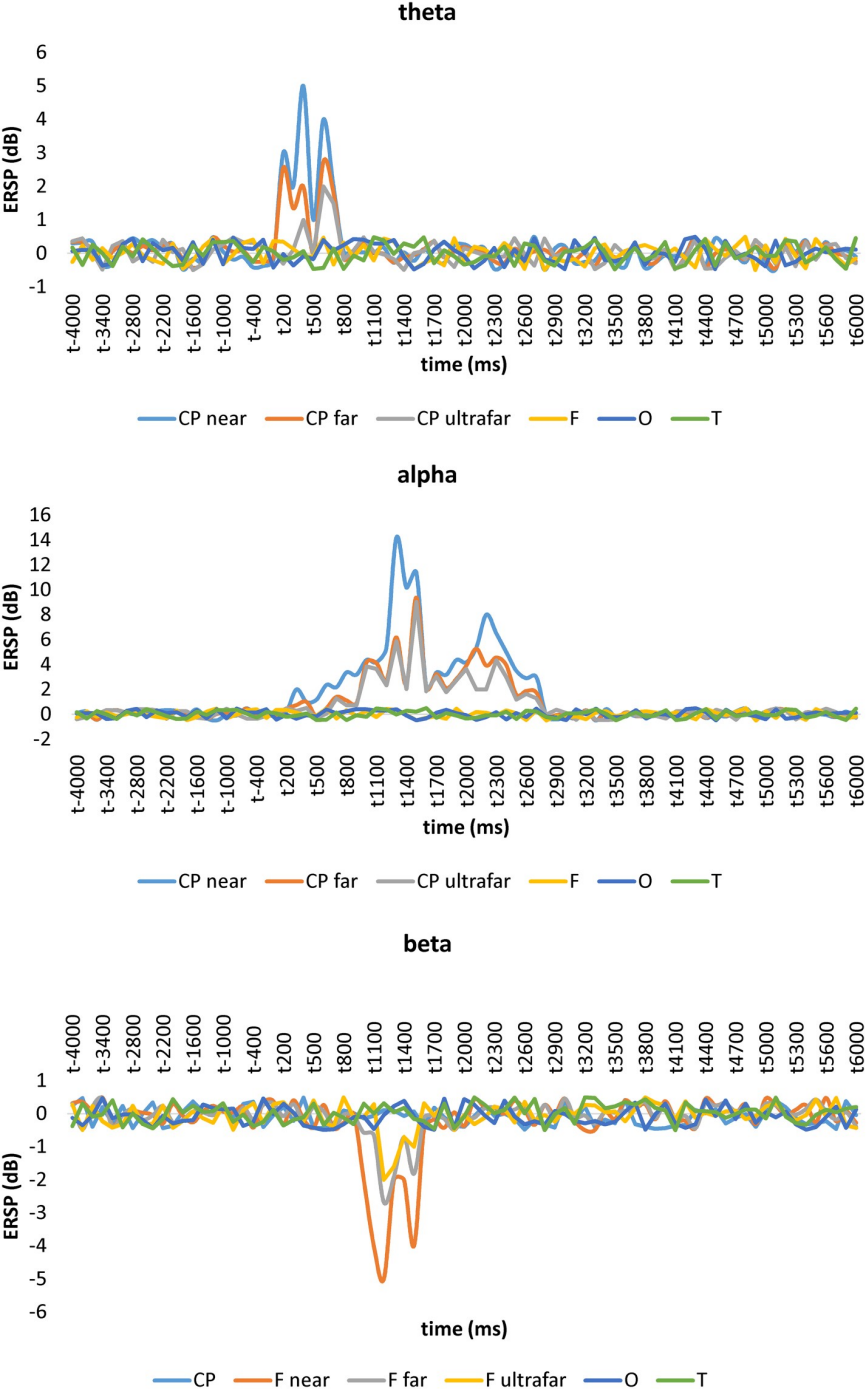
Event-related spectral perturbation (ERSP) time course across hand positions. ERSP are highlighted only for the significant electrode-groups.

We observed a significant post-stimulus frontal theta ERSP (t_(1,49)_ = -11, p<0.0001), a significant centroparietal alpha ERSP (t_(1,49)_ = -9, p<0.0001), and a significant frontal beta ERSP (t_(1,49)_ = 17, p<0.0001). Even though we focused on the general tendency of the ERSP in the post-stimulus period rather than the precise timing of the ERSP at different distances between the hand and the face, the near position induced a clear spectral perturbation within frontal electrodes in theta (100–700 ms) and beta frequency range (700-1500ms) compared to the far and ultrafar positions. On the contrary, we constantly found a centroparietal alpha ERSP starting soon after the electric stimulus at the wrist and progressively decreasing in magnitude up to the epoch end (100–2800 ms). As illustrated in Fig 3, theta and beta ERSPs were dependent on hand-to-face distance, i.e. ERSP was more evident at p2 (p = 0.005) and p1 (p = 0.006) compared to p0 in both of the frequency ranges. On the contrary, alpha frequency range was not dependent on hand-to-face distance (all comparisons p = 0.5).

To further characterize the post-stimulus spectral changes, we tested the *electrode-pool* × *hand-to-face-distance* ANOVA interaction for each of the six (dependent) variables obtained. Each of the features showed a significant effect of the factor electrode-pool (Table 1), whereas only the theta and alpha frequency range showed a significant effect of hand-to-face distance, parallel to the lack of hand-to-face distance effect on the ERSP magnitude.

**Table 1:** Significant ANOVA values.

		electrode-pool (F_(3,147)_)	hand-to-face-distance (F_(2,98)_)	electrode-pool× hand-to-face-distance (F_(6,294)_)
**Alpha**	**intercept**	F = 20 p<0.0001	p>0.2	F = 13 p<0.0001
	**slope**	F = 19 p<0.0001	p>0.2	F = 14 p<0.0001
	**quadratic**	F = 17 p<0.0001	p>0.2	F = 16 p<0.0001
**Beta**	**intercept**	F = 18p<0.0001	F = 16p<0.0001	F = 15 p<0.0001
	**slope**	F = 21 p<0.0001	F = 19 p<0.0001	F = 19 p<0.0001
	**quadratic**	F = 19 p<0.0001	F = 17 p<0.0001	F = 14 p<0.0001
**Theta**	**intercept**	F = 6.1 p = 0.004	F = 18p<0.0001	F = 13p<0.0001
	**slope**	F = 5.3 p = 0.001	F = 21 <0.0001	F = 11 <0.0001
	**quadratic**	F = 15 p<0.0001	F = 16 p<0.0001	F = 51 p<0.0001

ANOVA values (F, p) associated with each parameter (intercept, slope, and quadratic effect) in the three frequency bands (theta, alpha, and beta) for the principal effects of each factor (electrode-pool and hand-to-face-distance) and their interactions.

**Fig 4:**
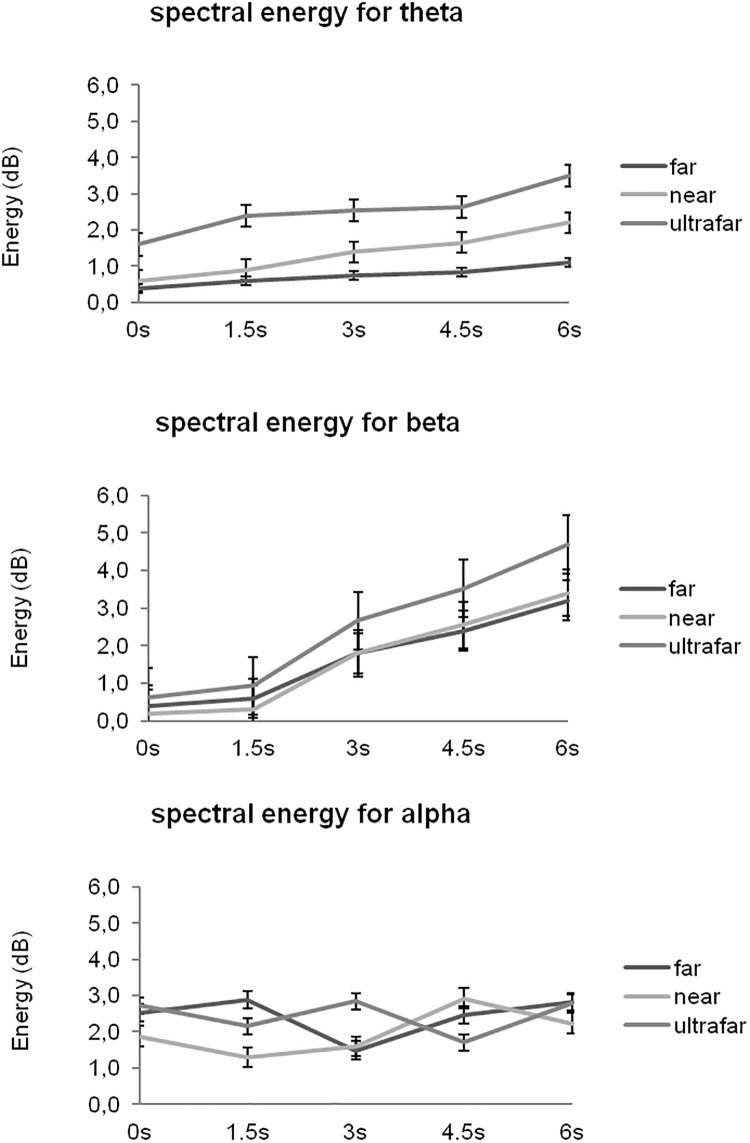
Evolution of the spectral energy based on the three parameters (intercept, slope, and quadratic effect) for theta, alpha, and beta band as a function of hand position. Vertical bars represent confidence intervals of 95% around the central value.

We also estimated the temporal evolution of the energy for each of the frequency bands (Fig 4). Overall, near position led to growing by epoch time and hand position-dependent energy magnitude (i.e., higher in near compared to far and ultrafar positions) only in the theta and beta bands.

All the ERSP features, ANOVA data, and feature data analyses related to the control experiment were non-significant with the exception of a centroparietal alpha ERSP (t_(1,24)_ = -9.9, p<0.0001). The features of such alpha ERSP were significant for the *electrode-pool* factor (intercept F_(1,24)_ = 25, p<0.0001; slope F_(1,24)_ = 111, p<0.0001; quadratic F_(1,24)_ = 177, p<0.0001), but not for the hand-to-face-distance factor and *electrode-pool × hand-to-face-distance* interaction.

## Discussion

Our data indicates that specific ERSPs within the frontal and centroparietal electrodes were elicited by the actual distance between the hand and the face. In particular, only theta and beta ERSPs were specifically dependent on the hand-to-face distance. Indeed, the near position induced a clear theta (early) and beta ERSP (later) within frontal electrodes compared to the far and ultrafar positions. On the contrary, we found a clear centro-parietal alpha ERSP that progressively decreased up to the end of the epoch. Last, we found a post-stimulus increase in all the ERSP coefficients.

Changes in sensorimotor rhythms normally occur during execution, observation, and imagery motor tasks, as well as during steady posture [74–77]. However, it is not completely understood whether these represent conscious processes, also with regard to PPS [77–82]. We suggest that the spatio-temporal perturbation of theta and beta frequency bands may be the marker of the top-down cognitive control that the frontal areas exert on the sensorimotor processes within the centroparietal areas occurring during a task execution [55–56,83–87]. Specifically, beta ERSP may be a marker of the purposeful motor preparation processes in relation to objects approaching the PPS [5,55–56,85]. In other words, beta oscillations may reflect the motoric “status quo”, that is, the dynamic remapping of motoric representation (of the PPS) as a function of the sensory-motor transformations required by the action kinematics in view of action execution [86–87].

On the other hand, the motor preparation-related cognitive processes, including working memory and attention, may be expressed by the theta ERSP [88–89]. The consequence of the frontal theta-beta perturbation was the long-lasting alpha ERSP, in which the magnitude was instead independent of hand-to-face distance. This response was also detectable in the control experiment not targeting the PPS, differently from theta-beta ERSPs, but it was substantially shorter and smaller in amplitude. Therefore, alpha ERSP may represent a PPS perturbation-related arousal phenomenon correlated with motor action preparation in response to the PPS-approaching target, even though it is not as specific as the theta-beta ERSP is [90–97].

Concerning the features of spectral perturbation, the post-stimulus increase of all the ERSP coefficients suggests a sustained mental activity during the post-stimulus period, thus proposing the involvement of an attentional load in the processing of the coupled visual (hand position) and sensory (electric stimulus) information [98–99]. The increase of the three ERSP coefficients suggests a shift of attention to facilitate the processing of stimuli, both attended and unattended [100–102].

Altogether, the spatio-temporal perturbation of the ERSPs and the modulation of the ERSP specific features suggest that the sensorimotor processing related to PPS may occur at a conscious level. This is further suggested by the potential entrainment of a frontoparietal networks overlapping with that subtending voluntary motor action and motor imagery and, partially, consciousness generation and maintenance [3,5,21,34,36,48,57,103]. Last, PPS activation may perturb the thalamo-cortical sensory gating mechanisms to facilitate sensory flow and increase cortical excitability to prompt motor responses. Therefore, theta-beta ERSP changes may express a higher level of cortical activation within frontoparietal networks related to the movements of the hand within the PPS and, likely, their conscious awareness thereof [63,104].

The specificity of the oscillatory changes we reported concerning PPS is suggested by the lack of specific EEG changes during the control motor task. We opted for such a control motor task because the actions targeting the extrapersonal space entrain brain networks (and thus evoke EEG changes) different from those entrained by PPS activation [7,68–71]. The lack of specific EEG changes during the control motor task also rules out the potential confounding effect of the diverse degree of required motor control and planning depending on the different hand positions, and of holding a certain posture as opposed to another, which might entail the activity of very different muscular patterns with a different effort [105].

### Limitations

We were unable to replicate the EEG power suppression usually observed during median nerve stimulation. In fact, nerve stimulation generates phase-locked and non-phase-locked responses within post-central (somatosensory) and pre-central (motor) cortices, respectively, which can be suppressed by muscle activation [63,95,106–108].

The lack of power suppression may depend on the partial uncertainty about the forthcoming hand position to be held from time to time (the sequence of hand position to be held was random) and the frequency of hand position change (ranging from 8 to 12 seconds). Moreover, the lack of power suppression may depend on a higher level of activation of the primary motor cortex owing to the perturbation of PPS compared to the motor task unrelated to the PPS [109].

Notably, we did not find any significant EOG response comparable to the hand-blink reflex responses. This was because we used much lower stimulation intensity than that necessary to evoke a hand-blink reflex, as we had to avoid any excessive sensory volley that could perturb the complex top-down control of subcortical networks. Further, the shorter frequency of electric stimulation may have induced hand-blink reflex habituation, which needs much slower stimulation frequency to be prevented (about 30 sec).

Last, we did not carry out validity checks to ensure that the sensory volleys were not influenced by peripheral factors. It may be the case that the changes observed in ERSP (known to have central generators) were due to alterations in afferent input because of transient variables, such as changes in posture, rather than to the sensory integration processes related to the PPS. To ensure this, it may be useful to investigate the N9 peak of somatosensory evoked potentials, which should differ by no more than ±10% pre- and post-stimulus trials [110]. However, we found significantly diverse ERSPs in the control experiment despite very similar motion sequencing. Thus, it seems unlikely a biasing effect on ERSP by alterations in afferent inputs.

### Conclusions

Our data indicate that PPS perturbation induces specific brain oscillations that varies with the hand-to-face distance changes. These oscillatory effects may represent the tonic and selective top-down modulation from the higher-order cortical areas (involved in the representation of the PPS) on sensorimotor circuits that oversee subcortical networks mediating the purposeful or reflex responses. Thus, there could be consciousness when PPS scenarios are mapped within frontoparietal areas in a fine-grained manner to continuously update the internal representations of the PPS to the end of preparing purposeful and/or reflexive motor responses. Once confirmed by larger sample studies and other methodological approaches (e.g., TMS-EEG, NIRS), the definition of the neural activity underlying PPS representation may be helpful to better characterize neurological conditions in which PPS can be impaired, as in the disorders of consciousness and cognitive decline.

## Supporting information

S1 Table Dataset.xls. Timed ERSP data are reported for each frequency band.
(XLS)
